# Taking Off the Rose-Colored Glasses: Experiences of Sexual Assault and Institutional Mistrust

**DOI:** 10.1177/08862605251363617

**Published:** 2025-08-22

**Authors:** Gabriella R. Petruzzello, Lucia F. O’Sullivan, Charlene F. Belu

**Affiliations:** 1 University of New Brunswick, Fredericton, Canada

**Keywords:** sexual assault, sexual violence, campus sexual assault, institutional betrayal, climate survey

## Abstract

Despite attempts to reduce sexual assault, approximately one-quarter of university students in Canada and the United States will experience a sexual assault during their undergraduate experience. In addition to the interpersonal trauma survivors experience during a sexual assault, institutions may respond in ways that amplify this trauma, failing students at an especially vulnerable point. Sexual assault has a long-term impact on the way survivors perceive the world and interact with institutions, often resulting in disillusionment. This study sought to understand how experiencing sexual assault, including the timing of that assault, is linked to university students’ perceptions of institutions and their policies, perceived risk of sexual assault, and rape myth acceptance. Students from a Canadian institution (*N* = 1,220) completed an online survey that assessed their sexual assault history, from which they were grouped in terms of timing of the assault experience: pre-university, during university, prior to and during university, and no sexual assault history. A one-way multiple analysis of variance revealed that individuals with any sexual assault history reported lower institutional trust, more negative perceptions of their institution’s sexual assault policies, greater perceived risk of sexual assault, and lower endorsement of rape myths than those with no sexual assault history. Differences from those with no assault history were larger among those who had experienced sexual assault during their university experience. Additional exploratory analyses showed that diminished campus belonging among survivors of sexual assault was mediated by lower levels of institutional trust. Implications are centered around the long-term impacts of sexual assault, the role that institutions play in amplifying negative outcomes, and the role that these impacts play in reducing reporting and help-seeking.

Despite increased awareness and attempts to reduce sexual assault on college campuses, prevalence rates have remained largely the same, with an estimated 25% of U.S. and Canadian college students experiencing a sexual assault during their undergraduate experience (Basile et al., 2022; Fedina et al., 2018; MacKenzie, 2019; Mellins et al., 2017; Muehlenhard et al., 2017). The way that institutions respond following an incident of sexual violence has a protective or deleterious impact on survivors’ health, well-being, and organizational connection (Adams-Clark et al., 2026; Smidt et al., 2023). In spite of extensive efforts by institutions to address the high prevalence of sexual assault, students often perceive their institutions as failing to provide adequate prevention education and victim support (Garcia et al., 2012; Marques et al., 2020). Only 50% of U.S. students believed their institution would conduct fair investigations into campus sexual assaults (Cantor et al., 2017), and students were more likely to report to police than university officials, regardless of whether the assault happened on campus (Moore & Baker, 2018; Richards, 2019). In both the United States and Canada, few assaults result in a formal complaint to the university, rarely does an investigation produce a decision regarding culpability, and almost no investigations end with perpetrator suspension or expulsion (Lee & Wong, 2017; Richards, 2019).

Even with the critical influence that university response has on student outcomes following sexual assault (Adams-Clark et al., 2026; Smidt et al., 2023), surprisingly little is known about impaired trust in these institutions by survivors of sexual assault, including views about safety and policy in place to support survivors of sexual assault. Interpersonal and institutional trust are components of positive well-being and belonging (Poulin & Hasse, 2015; Zhao et al., 2024) and sexual assault can deteriorate such trust and increase beliefs that promote mistrust and awareness of systemic problems (Dean, 2021; Herres et al., 2021; Neilson et al., 2018). Survivors may break from the default sense of optimism or trust in institutions (taking off the rose-colored glasses) as a function of their experiences, no longer seeing institutions as safe havens in a crisis, but as contributors to systemic injustice, further amplifying and extending their trauma (Smith & Freyd, 2013). Given how many young people experience sexual assault while attending university, this study advances work in this area by investigating experiences of assault and links to institutional trust, including perceptions about institutional climate, evaluation of sexual assault policy, general sense of safety, and rape myth acceptance (RMA).

## Institutional and Attitudinal Impacts of Sexual Assault

For many, sexual assault is an acutely traumatic experience, often made worse by university structures that fail to adequately support individuals. These traumatic experiences can change how individuals connect to and feel about institutions and their social environment, although the literature on the links between sexual assault history and impaired institutional connection is notably limited to date (Firkey et al., 2023; Gezinski et al., 2025; Herres et al., 2021). A number of factors in this realm have been identified that may be related to sexual assault in the campus context. These include feelings of belonging, trust in one’s institution, sense of safety, and RMA.

### Belonging

Campus belonging is defined as a student’s perception that they are an integral part of the campus community through shared values, goals, and experiences (Strayhorn, 2018). It is a central focus of many recruitment and retention efforts, and universities spend considerable time, effort, and funding to reinforce belonging as a way to help ensure student success and well-being (Greenland & Moore, 2021). Campus belonging drives academic success and is a key predictor of physical and psychological well-being (Begen & Turner-Cobb, 2015; Gopalan & Brady, 2020). Individuals who report betrayal by their university following sexual violence also tend to report less belonging in the form of decreased attachment to their university (Rosenthal et al., 2017). Existing research tends to show that survivors of campus sexual assault report lower perceived campus belonging and campus community safety, which is associated with increased academic challenges and mental health, and alcohol use problems (Firkey et al., 2023; Gezinski et al., 2025). Inversely, campus belonging can buffer negative psychological consequences, including negative post-traumatic stress disorder (PTSD) symptoms following sexual assault (Bilal & Herres, 2025). Alternatively, research by Herres et al. (2021) did not find a relationship between experiencing campus sexual assault and perceived sense of community. This research demonstrates the potential need for more nuanced investigations of the links between sexual assault history and campus belonging.

### Institutional Trust

Positive perceptions of institutions are critical for societal trust, functioning, and mental health (Spadaro et al., 2020). Experiencing sexual assault is linked to deteriorated trust in one’s institution regarding its response to incidents of sexual assault. Betrayal by one’s institution exacerbates existing trauma and breeds mistrust, as individuals find danger in a place where they had previously expected to find safety (L. S. Brown, 2021; Smith & Freyd, 2014). Trust in one’s institution is important for those who experienced sexual assault as it is a consistent predictor of reporting to both police and university officials, which can help to increase the safety of the survivor and their surrounding community (Holland, 2020; James & Lee, 2014; Moore & Baker, 2018; Mushonga et al., 2020; Rizzo et al., 2021). College students with more negative perceptions of campus supportiveness, college responsiveness, and college handling of sexual assault were more likely to have had an experience of campus sexual assault, although the directionality of this association is unclear (Herres et al., 2021). In line with these findings, LGBT university students, who have a greater risk of experiencing sexual violence, are more likely to report distrust in the university and police reporting and response processes (Oberweis et al., 2025). Findings of a study by Dean (2021) using a university sample support the view that experiencing campus sexual assault or knowing a friend who has is related to more negative perceptions of institutional processes, including heightened perceptions of a rape culture on campus, feeling as if students do not receive enough information about sexual assault, and a lower likelihood of reporting a future sexual assault. Furthermore, a longitudinal study with university survivors of sexual assault found that expecting other people, such as campus administrators and one’s university, to react negatively to sexual assault disclosures contributed to risk for future revictimization (Miller et al., 2011). This longitudinal study shows not only the impact of sexual assault history on perceived stigma but also the negative outcomes that can stem from such mistrust.

### Perceived Risk

In line with elevated risk of sexual violence, research spanning decades has demonstrated that women have a greater perceived risk of crime than do men (see Collins, 2016 for a review), likely due to what is called the shadow of sexual assault whereby women are socialized to fear being targeted by aggressive men (Ferraro, 1996; Mellgren & Ivert, 2019). Relatedly, survivors of sexual assault tend to have higher perceived sexual assault risk and lower sense of overall safety than non-victims (A. L. Brown et al., 2005; Culbertson et al., 2001; Neilson et al., 2018). Although this elevated perception of risk is warranted given women’s heightened risk of sexual assault in general and the heightened risk for survivors to experience subsequent sexual trauma, the constant sense of unease and frequent fear generated by perceptions of that risk are related to lower subjective well-being and heightened psychological distress (Sulemana, 2015; Watson et al., 2015). Understanding the extent to which survivors of sexual assault feel at risk for revictimization in a range of contexts has important implications for mitigating long-term psychological outcomes that stem from constant vigilance.

### Rape Myth Acceptance

RMA, or endorsement of stereotypes and false beliefs about sexual violence, has been a heavily researched area with regard to attitudes toward social violence. Results are mixed regarding the influence of sexual violence history on RMA. Some studies have found lower RMA in survivors of sexual assault (Lathan et al., 2023; McMahon, 2010; Navarro & Tewksbury, 2017; Vonderhaar & Carmody, 2015), whereas other studies have found no relationship (Fansher & Zedaker, 2020; Powers et al., 2015) or elevated RMA (PettyJohn et al., 2023). Survivors of sexual assault tend to accept rape myths at lower levels, which may be due to personal experiences that challenge, counter, or change these myths into more adaptive beliefs as a coping mechanism to reduce self-blame and encourage healing. Research has further demonstrated that RMA is positively related to perceptions of institutions (Dean, 2021), possibly because endorsement of rape myths ultimately places the blame for sexual assault on the survivor rather than institutions or the perpetrator. While individuals who reject rape myths tend to hold perpetrators accountable for their actions, they also may be more likely to hold institutions accountable for the cultures they create and the damaging consequences of these environments. Lower levels of RMA have important implications for the likelihood of reporting and willingness to use campus services (Holland, 2020; Lathan et al., 2023). In sum, RMA appears to be influenced by sexual assault history and plays a potential role in institutional trust.

## The Current Study

This study sought to determine whether perceptions of campus belonging, institutional trust, perceived sexual assault risk, and RMA differ among individuals who experienced sexual assault *prior* to their university experience, individuals who experienced sexual assault *during* their university experience, individuals who experienced sexual assault *both during and prior* to their university experience, and individuals with *no* history of sexual assault. Short of a longitudinal design, this methodology allows investigation of the directional relationship between victimization and institutional mistrust by examining whether experiencing sexual assault is generally associated with negative institutional perceptions, perceived risk, and RMA, or if these associations are unique to individuals who experienced sexual assault in the context of attending their institution. Overall, this study advances the literature by demonstrating how sexual assault, including the timing of that assault, is linked to perceptions of institutions, perceived risk of sexual assault, and RMA among study participants.

This study also briefly situated these findings in the context of low reporting rates and service use as well as non-use among those who experienced sexual assault. Based on previous research, we hypothesized that the individuals with a campus sexual assault history would report lower campus belonging, lower institutional trust, more negative perceptions of institutional sexual assault policies, higher perceived sexual assault risk, and lower RMA compared to individuals without a campus sexual assault history. As an exploratory hypothesis, we predicted that individuals with any type of sexual assault experience prior to university would report similar results on these variables to those who experienced sexual assault while at university, which would provide support for a broader relationship between sexual assault experiences and the study variables.

## Method

### Participants

Participants (N = 1,294) were recruited from a campus community in eastern Canada. Eliminating participants who failed validity checks (*n* = 74) resulted in a final sample of 1,220 participants (see Table 1). Participants were on average 23 years of age (*SD* = 6.07) and full-time students (92.3%, n = 1,123). Participants were primarily in undergraduate (78.3%, *n* = 956) or graduate/professional degree programs (19.7%, *n* = 240). There was a higher percentage of women (65.7%, *n* = 801) than men (32.9%, *n* = 401) or those identifying as transgender (1.3%, *n* = 17). Although most participants identified as heterosexual (81.7%, *n* = 995), a sizable percentage identified as part of the LGBQ+ community (12%, *n* = 147). The sample was predominantly White (85.7%, n = 1,045), with an additional 6.6% identifying as Asian (*n* = 81). Each of the other racial groups comprised less than 3% of the sample. Regarding relationship status, 37% were in a committed relationship (*n* = 452), 26.2% were not currently in a relationship (*n* = 320) 15.7% were casually dating (*n* = 191), 9.9% had never been in a relationship (*n* = 121), and 8.3% were married (*n* = 101).

**Table 1.** Sample Demographic Characteristics.

Characteristics	%	*n*
Gender/sex		
Female	65.7	801
Male	32.9	401
Transgender female	0.3	4
Transgender male	0.2	3
Other	0.8	10
Sexual orientation		
Heterosexual	81.7	995
Bisexual	8.3	101
Gay or lesbian	2.9	36
Asexual	0.8	10
Questioning	1.3	16
Don’t know	0.7	8
No labels preferred	2.5	31
Other	1.7	21
Race/ethnicity		
Caucasian/White	85.9	1,045
Asian	6.6	81
Multiracial	2.3	28
First nations	1.6	19
African Canadian/Black	1.3	16
Arab	0.4	5
Metis	0.4	5
Other	0.7	8
Year of degree		
First year undergraduate	11.2	136
Second year undergraduate	20.0	243
Third year undergraduate	19.6	238
Fourth year undergraduate	20.3	246
Fifth year undergraduate	7.7	93
Graduate or professional degree	19.8	240
Other	1.5	18
Relationship status		
In a committed relationship	37.2	452
Not currently in a romantic relationship, but have been before	26.3	320
Casually dating	15.7	191
Never been in a relationship	10.0	121
Married	8.3	101
Other	2.5	31
Place of residence		
Off-campus in a house or apartment	81.2	982
On-campus in a residence	18.8	227
With whom they lived		
Roommate(s)	30.1	366
Family	24.9	302
Romantic partner	17.7	215
Alone	16.5	201
Friend(s)	10.5	128
Other	0.2	3

*Note.* Total *N* = 1,220.

### Measures

Data from the current study were part of a larger online survey pertaining to campus climate around issues of sexual assault (see also O’Sullivan et al., 2023). Study questionnaires are available on Open Science Framework (osf.io/3jsrg) and were informed by the White House Task Force to Protect Students from Sexual Assault (2014), which provides guidance on best practices for campus climate surveys to accurately assess the nature and prevalence of sexual violence in a university context. Items were compiled from the 2014 White House Task Force report to construct survey instruments in conjunction with other relevant resources (cited in each subsection) that addressed campus belonging, institutional trust, and perceived risk of sexual assault.

#### Victimization History

Victimization history was assessed using two items: “Have you been sexually assaulted since you have been a student at (institution name)?” and “Before attending (institution name), did you experience any of these unwanted forms of sexual contact?” For both questions, a definition of sexual assault was provided as “being unwilling to engage in or not giving consent to sexual contact, including touching of a sexual nature, oral sex, vaginal sex, anal sex, or vaginal or anal penetration with an object, or by a body part other than a penis or a tongue.” Providing a definition of the broad spectrum of behaviors that constitute sexual assault and the definition of consent was used to promote disclosure and increase accuracy of responses (Abbey et al., 2005; Koss et al., 2007). Response options included yes, no, unsure, or other. These responses allowed us to form the comparison groups centered around the type of victimization history. Individuals who responded yes to having experienced sexual violence while at the university, but not before the university, were categorized as in the During-University group. Similarly, individuals who had experienced sexual assault prior to attending the university (but not during) were categorized as in the Pre-University group. Those reporting yes or no to both questions comprised the Both Prior to and During-University group or the No Victimization History group, respectively. Individuals who responded “Unsure” or “Other” were not included.

#### Use of Sexual Assault Resources

Participants who reported experiencing campus sexual assault were prompted with a list of campus and community resources and asked which resources they had used (if applicable) to discuss their experiences of sexual assault, with the option to select multiple resources. These options included campus counseling services, campus security, the university health center, the local sexual assault center, a non-university mental health practitioner, or a non-university family doctor. Participants could also indicate whether they chose not to disclose the sexual assault. Using the same list, participants reported if there were any places or resources that they had wanted to visit to discuss their experience of sexual assault, but for some reason had opted not to visit.

#### Campus Belonging

Campus climate, including school connectedness and belonging, was measured using 12 items, including 7 from the Brief Sense of Community Scale tapping into the constructs of needs fulfillment, membership, influence, and emotional connection (Peterson et al., 2008). Five additional items were added to measure campus safety, concern for welfare, respect for student concerns, fair treatment, and happiness to be a part of the university (White House Task Force to Protect Students from Sexual Assault, 2014). All study questionnaires were rated on a 5-point scale from *Strongly Disagree* (1) to *Strongly Agree* (5) unless otherwise specified. The items demonstrated high internal consistency in the current study (α = .91).

#### Institutional Trust

##### Trust in the Institution

Institutional trust stems from the belief that systems designed to support and protect can be relied upon in times of need. Trust in the institution’s ability to support students who have experienced a crisis was measured using five items adapted from the six-item validated Trust in the College Student Support System Scale (Sulkowski, 2011; White House Task Force to Protect Students from Sexual Assault, 2014). This measure assessed students’ trust in various campus resources to manage crises effectively (e.g., “If a crisis happened on campus, (insert university) would handle it well”) and promote campus safety. The two questions about campus administrators and campus security were combined into one question prior to data collection, reading “University officials (administrators, campus security) at (institution name) do enough to protect the safety of the students.” This measure indicated high internal consistency (α = .86) in the current study and has shown good internal consistency and convergent and discriminant validity in previous research (Sulkowski, 2011).

##### Perceptions of Sexual Assault Leadership, Policies, and Reporting

Perceptions of campus processes related to reporting were measured using seven items from the White House Task Force to Protect Students from Sexual Assault (2014). These items started with “If someone were to report a sexual assault to a campus authority, how likely is it that. . .,” and were used to capture the perceived likelihood that the university would enact supportive policies and reporting practices specifically pertaining to sexual assault response. All items were on a 5-point scale from *Very Unlikely* (1) *to Very Likely* (5). Example items include “(institution name) would take the report seriously?” and “(institution name) would take steps to protect the safety of the person making the report.” These seven items demonstrated a high internal validity (α = .92) in the current study.

#### Perceived Sexual Assault Risk

Perceived sexual assault risk was measured using a brief four-item measure that captures the precontemplation stage of change (Levesque et al., 1999; see also Higher Education Data Sharing Consortium, 2024) where individuals are not aware of a problem or perceive the problem to have only minimal risk, including “I believe that the number of sexual assaults that occur at (institution name) campus is low,” and “I do not believe that I am, or any of my friends are, at risk for being sexual assaulted on the campus of ___.” The Cronbach alpha was .90 for the current study.

#### Rape Myth Acceptance

The Illinois Rape Myth Acceptance Scale–Short Form (Payne et al., 1999) is a widely adopted measure used to assess endorsement of rape-supportive and justifying beliefs. The short form has strong psychometric properties, including a high internal reliability (Payne et al., 1999). The original short form included 17 items assessing 7 categories of rape myths (e.g., “She asked for it,” “It wasn’t really rape”). In this study, the filler items were omitted, and a one-question item was added to reflect an eighth myth that “Men cannot be sexually assaulted because sexual assault only happens to women,” a construct with decades of research support (Struckman-Johnson & Struckman-Johnson, 1992; Walfield, 2021). The Cronbach alpha indicated strong internal consistency in the current study (α = .94).

### Procedure

All full- and part-time students at one institution in eastern Canada received an email invitation and reminder emails from the university registrar’s office, with a survey link for the current study leading to the anonymous survey platform Qualtrics, where no names or identifying information were collected. Further recruitment announcements were posted online, including through student electronic news, the university’s social media pages, and the university newspaper. All participants completed an informed consent protocol in advance of receiving the survey. Participants were subsequently debriefed with resources and had the opportunity to enter a gift card raffle. All study procedures were approved by the authors’ ethics review board.

### Data Analysis

A one-way multiple analysis of variance (MANOVA) was used to examine differences in campus belonging and institutional trust, perceptions of university sexual assault policies, perceived sexual assault risk, and RMA across the four groups who varied in sexual assault history. Participants were categorized into four groups based on the timing of their sexual assault experiences: Pre-University Victimization, University Victimization, Both Pre- and During-University Victimization, and No Victimization. Descriptive statistics are presented for sexual assault prevalence by gender identity and timing of sexual assault. Frequencies for reporting and for actual and desired use of campus and community resources are additionally shown.

## Results

The sample reported a high rate of sexual assault in their histories with 26.7% (*n* = 326) of students reporting an incident of sexual assault prior to attending the university, 4.7% (*n* = 57) reporting a sexual assault while attending the university (although not necessarily associated with the university), and another 5.6% (*n* = 68) reporting sexual assault both prior to and during their university experience. Overall, over one-third (37%, *n* = 451) of students reported having experienced at least one incident of sexual assault in the past. The majority (56.6%, *n* = 691) of the sample reported no sexual assault history. A small proportion of participants responded unsure or “other” to the victimization history questions and were not included in subsequent analysis (6.4%, *n* = 78).

Of the 381 male participants who responded to the victimization items, 13.4% (*n* = 51) reported an incident of sexual assault prior to university, a small percentage (1.8%, *n* = 7) reported a sexual assault while attending the university and 2.4% (*n* = 9) reported experiencing a sexual assault both prior to and during their university experiences. In total, 17.6% (*n* = 67) of male participants reported having experienced at least one incident of sexual assault in the past. Of the 746 female participants who responded to the victimization items, 36.1% (*n* = 269) reported an incident of sexual assault prior to university, 6.7% (*n* = 50) reported a sexual assault while attending the university, and 7.5% (*n* = 56) reported a sexual assault both prior to and during university. Over half (50.3%, *n* = 375) of female participants reported at least one past incident of sexual assault. Of the 14 transgender or non-binary individuals who responded to the victimization items, 5 (35.7%) experienced a sexual assault prior to university, and 3 (21.4%) reported experiencing a sexual assault both prior to and during university. Over half (57.1%) experienced at least one past incident of sexual assault.

Accessing campus and community reporting resources occurred at low levels (1.6%) among the individuals who experienced campus sexual assault. Only 2 of the 125 individuals formally reported their assault (either on campus or in the community) and both described these procedures as entirely unhelpful. Individuals sought out a range of resources to discuss their experiences of sexual assault, with the most common being the university counseling services (20%, *n* = 25), a mental health practitioner (6.4%, *n* = 8), the university health center (4.8%, *n* = 6), or a family doctor (4.8%, *n* = 6). Only 4.8% (*n* = 6) and 2.4% (*n* = 3) utilized the local sexual assault center and campus security, respectively. Notably, 39% (*n* = 49) of individuals did not discuss their experiences of sexual assault with anyone. Consistent discrepancies were apparent between the number of individuals who used the services (actual use) and the number of people who wanted to use a service (desired use) but did not following an assault. Nearly an additional quarter of survivors wanted to use the university counseling services (24.8%, *n* = 31), followed by 28.8% (*n* = 36) of survivors equally split between wanting to use the university health center or the local sexual assault center. Approximately one-third wanted to see a mental health practitioner (12.8%, *n* = 16), their family doctor (10.4%, *n* = 13), or campus security (14.4%, *n* = 18).

A first analysis tested our hypotheses that individuals with a campus sexual assault history (and potentially even those with any sexual assault history) would report lower campus belonging, lower institutional trust, more negative perceptions of institutional sexual assault policies, higher perceived sexual assault risk, and lower RMA compared to individuals without a sexual assault history. A one-way MANOVA was conducted to assess differences among the four groups who varied in sexual assault experience (Prior to University; During University; Both Prior to and During University; No Experience). The dependent variables were campus belonging, trust in university support system, perceptions of leadership, policies and reporting, perceived sexual assault risk, and RMA.

A main effect of experience emerged on the omnibus test, Pillai’s Trace = 0.16, *F*(15, 3354) = 12.87, *p* < .001, ${\eta_{\rho }}^{2}$ = .054. Individuals with a history of sexual assault tended to differ in their attitudes compared to individuals with no history of sexual assault. Univariate analyses confirmed this main effect of experience for all dependent variables. Three of the dependent variables had a small effect size (campus belonging: ${\eta_{\rho }}^{2}$ = .008, trust in university support system: ${\eta_{\rho }}^{2}$ = .042, perceptions of leadership, policies and reporting: ${\eta_{\rho }}^{2}$ = .055, and RMA: ${\eta_{\rho }}^{2}$ = .048) and one had a medium effect size (perceived risk of sexual assault: ${\eta_{\rho }}^{2}$ = .148). Descriptive statistics and correlations for study variables are shown in Table 2. Tukey post hoc tests at .05 were performed for each of the significant dependent variables.

**Table 2.** Descriptive Statistics for the Variables in the Study.

Variable		*M*	*SD*	1	2	3	4	5
1	Campus belonging	3.71	0.64	—				
2	University trust	3.37	0.77	.48**	—			
3	University policy perceptions	3.72	0.79	.41**	.68**	—		
4	Perceived sexual assault risk	3.07	1.03	−.18**	−.48**	−.52**	—	
5	Rape myth acceptance	1.43	0.55	.01	.17**	.15**	−.37**	—

*Note.* *N* = 1,220. All items were on a 5-point scale. *SD* = standard deviation.

** *p* < .01.

As shown in Table 3, individuals did not differ on their level of campus belonging based on victimization experience, despite a significant effect at the univariate level. Trust in the university student support system was higher for individuals with no history of sexual assault compared to those who experienced sexual assault prior to university, during university, and both prior to and during university. Individuals who experienced sexual assault prior to university had greater trust than the individuals who experienced sexual assault both prior to and during university. For perceptions of leadership, policies, and reporting, individuals with no history of sexual assault rated the campus as more supportive than those who experienced sexual assault prior to university, during university, and both prior to and during university. Experiencing sexual assault prior to university was associated with more positive perceptions of university sexual assault policies compared to individuals who experienced sexual assault during university or both prior to and during university. Regarding perceived risk of sexual assault, individuals with no history of sexual assault reported significantly lower perceived risk than their counterparts who had experienced sexual assault prior to university, during university, and both prior to and during university. Those who experienced sexual assault prior to university reported lower perceived risk than the two university groups. Finally, acceptance of rape myths was higher among individuals with no history of sexual assault compared to individuals who experienced sexual assault prior to university, during university, or both prior to and during university.

**Table 3.** Descriptive Statistics by Type of Sexual Assault Experience.

Variable	No Experience (*n* = 683)		Pre-University (*n* = 317)		During University (*n* = 57)		Both Prior and During University (*n* = 67)	
	*M*	*SD*	*M*	*SD*	*M*	*SD*	*M*	*SD*
Campus belonging	3.75	0.63	3.65	0.63	3.60	0.77	3.59	0.68
University trust	3.47_abc_	0.74	3.30_ad_	0.76	3.08_b_	0.86	2.89_cd_	0.90
University policy perceptions	3.85_abc_	0.75	3.61_ade_	0.77	3.28_bd_	0.84	3.30_ce_	0.86
Perceived sexual assault risk	2.79_abc_	0.96	3.33_ade_	1.00	3.93_bd_	0.76	4.02_ce_	0.85
Rape myth acceptance	1.51_abc_	0.60	1.32_a_	0.44	1.17_b_	0.19	1.20_c_	0.30

*Note. N* = 1,124. Means with the same subscript differ significantly at the *p* < .05 level. *SD* = standard deviation.

To better understand the mixed results of the significant omnibus and univariate tests, but no mean differences for the campus belonging variable, exploratory mediation analyses were conducted to determine if lower campus belonging was associated with sexual assault history through its relationship to institutional trust variables. Results demonstrated that the relationship between sexual assault history and campus belonging was mediated through institutional trust variables (see Supplemental Material for more information).

## Discussion

This study provides the first insights into our knowledge of the ways in which both campus sexual assault and sexual assault experiences in general are linked to impairments in campus belonging, institutional trust, perceived safety, and rape myth endorsement. Overall, survivors of sexual assault reported lower institutional trust, more negative perceptions of institutional sexual assault policy, heightened perceptions of sexual assault risk, and lower RMA, with variability in the results based on the institutional context of the assault. We found low rates of reporting to the university or police and negative experiences for the few who used the reporting services. Survivors often sought support through other resources for counseling or medical support, although there was still a portion of these survivors who experienced additional barriers to such services. In short, most suffered in the aftermath of their assault while trying to persist in an environment clearly viewed as indifferent, incompetent or unsympathetic.

Campus belonging, a key predictor of overall health and well-being for students (Begen & Turner-Cobb, 2015; Gezinski et al., 2025; Rosenthal et al., 2017), was related to sexual assault history, but only through its relationship with institutional trust. The discrepancy between the significant univariate effect of belonging and the insignificant mean differences points to a small size of this relationship and the overlap between the outcome variables of institutional trust and campus belonging that was illustrated only through a model measuring indirect relationships. These results mirror findings from Herres et al. (2021) showing the absence of a direct relationship between experiencing campus sexual assault and lower campus belonging, but the presence of a relationship between campus sexual assault and lower perceived college responsiveness, which in turn was positively correlated with lower campus belonging. This result indicates that survivors were more likely to distrust their institution and its sexual assault policies, which contributed to lower feelings of campus belonging.

In contrast to the indirect relationship between sexual assault history and campus belonging, there was a broad and direct relationship exhibited between institutional trust, perceptions of campus sexual assault policies, and any form of sexual assault history. Individuals with no history of sexual assault had more confidence in their university’s ability to handle crises effectively and rated the campus’ sexual assault policies as more effective and supportive compared to individuals who experienced sexual assault, regardless of whether the assault occurred during or prior to their university experience. Notably, this relationship was stronger for individuals who had experienced sexual assault during their university experience as compared to individuals who had experienced sexual assault prior to university. Experiencing sexual assault in an institutional context like a university creates the opportunity for the institution to let the survivor down, as reflected by the increased mistrust associated with experiencing sexual assault in an institutional context (Dean, 2021; Herres et al., 2021; Sall & Littleton, 2022). This finding replicates longitudinal results showing that survivors of campus sexual assault tend to exhibit mistrust (also referred to as stigma threat), expecting inadequate or harmful responses to their sexual assault disclosure (Miller et al., 2011). Of grave consequence, expecting these negative responses to disclosure is related to maladaptive coping and later victimization which warrants the development of strategies to mitigate these harmful outcomes.

In addition, these results illustrated a general relationship between experiencing sexual assault and elevated institutional mistrust. Because individuals who experienced sexual assault outside of the university context also reported mistrust in institutional policies, this research provides preliminary evidence that experiencing sexual assault, regardless of whether it occurred in a university context, is associated with more negative perceptions of institutions and their abilities to care for those under their charge. This elevated mistrust and dissatisfaction among survivors is reflected in research showing broad dissatisfaction with institutional policies and responses to sexual assault (Cantor et al., 2017; Garcia et al., 2012). These findings reiterate the theme that experiencing sexual assault takes away the optimistic lens (aka rose-tinted glasses) many people have regarding the institutions with which they interact, with this clash yielding a sense of significant mistrust and uncertainty.

Perceived risk of sexual assault was understandably elevated for individuals with a history of sexual assault compared to individuals with no history of sexual assault. These differences were particularly salient among individuals who experienced sexual assault in the university context, although experiencing sexual assault outside of the university context was also associated with perceived risk. These results replicate past research that survivors of sexual violence feel they are at a greater risk of future victimization and feel a greater sense of danger (A. L. Brown et al., 2005; Neilson et al., 2018). They also comprise preliminary evidence that survivors of campus sexual assault feel especially at risk of victimization in their university environment, which can amplify negative outcomes like academic challenges following experiences of sexual violence (Gezinski et al., 2025).

RMA appeared to be broadly associated with sexual assault history regardless of whether the sexual assault occurred in a university context. Lower levels of RMA among survivors of sexual violence have been supported by existing research (Lathan et al., 2023; Navarro & Tewksbury, 2017) and have practical implications including greater likelihood of reporting and willingness to use campus services (Holland, 2020; Lathan et al., 2023). These results offer novel evidence that lower rape myth endorsement among survivors of sexual violence does not differ as a function of institutional context.

### Limitations and Future Directions

The findings from this study showed that survivors of campus sexual assault had higher levels of institutional mistrust, but it remains unclear if institutions themselves are contributing to these relationships. It could be that the temporal closeness of the sexual assault is contributing to this elevated mistrust, and that differences between the pre-university and university sexual assault groups are due to the additional time to heal and emotionally recover following the event. Furthermore, it is uncertain whether the elevated institutional mistrust was indicative of institutional betrayal or the general negative psychological symptoms that stem from this kind of interpersonal trauma. Along these lines, it is possible that elevated mistrust was a result of negative experiences with university services or reporting and feeling as if the university did not respond in a supportive way, but it is also possible that the social withdrawal and depression following sexual assault contributed to these trends. A longitudinal design would allow for a better understanding of temporal precedence.

Another limitation is the dichotomous classification of sexual assault experiences as either having happened or not. While this allows for easy classification and assignment into groups based on experiences, significant nuance about the range of sexual assault experiences is lost by relying on such a dichotomous approach. Also, non-equivalence was a limitation in the current study as the victimization measure assessing pre-university sexual assault used behavior-based language similar to the Sexual Experiences Survey (Koss et al., 2007), whereas the during-university sexual assault question specifically asked participants to identify if they had experienced sexual assault while at the university. Asking participants to label their experiences as sexual assault often results in underreporting, since individuals do not consider their experiences as severe enough to constitute sexual assault (Papp & McClelland, 2021). Though a broad definition was provided for behaviors that constitute sexual assault, individuals may still feel as if their experience did not meet the criteria. On the other hand, the results demonstrating the effects of pre-university victimization on institutional trust and support for institutional policies may actually be underestimated given the inclusion of behaviors that are lower in perceived sexual assault severity. Future research is warranted with more nuanced methodology to estimate experiences of sexual violence within and outside the institutional context. Such research could also best incorporate measures assessing time since sexual assault to gain insight into the contextual factors shaping trauma response outcomes.

This study warrants future research to elucidate further the nature of institutional mistrust. Given the relationship between campus belonging and a host of positive outcomes (Begen & Turner-Cobb, 2015), future research is needed to determine if decreases in campus belonging associated with lower institutional trust correspond to negative experiences seeking support from one’s university or if these declines are a result of the traumatic event that occurred within the university context. In addition, qualitative research could allow for a greater understanding of how survivors perceive their university following a campus sexual assault, and how these perceptions differ among survivors experiencing varying levels of institutional betrayal. Whereas this study primarily looked at group-based differences, future research could examine predictors of campus belonging, institutional betrayal, perceived risk, and RMA as a function of assault characteristics, experiences of institutional support or betrayal, and time from current assault. Furthermore, a predictive model could help determine vulnerable populations and the relative influence of assault characteristics versus institutional characteristics in determining long-term outcomes. Other promising avenues for future research could specifically examine how experiencing sexual violence shapes perceptions of safety within relationships, broad levels of pessimism and optimism, and beliefs about justice and fairness.

### Implications

The current study makes clear that survivors who report higher institutional mistrust are more likely to experience a host of negative physical and psychological outcomes (Poulin & Hasse, 2015; Zhao et al., 2024) and may be less likely to engage in reporting processes and use campus services (Holland, 2020; Mushonga et al., 2020). Indeed, the rates of reporting were very low in this sample, and service use tended to be minimal, in line with existing research (James & Lee, 2014; Moore & Baker, 2018). Although some survivors sought out services like counseling, most survivors experienced significant barriers to service use.

Even individuals who experienced sexual assault before university reported notable levels of mistrust of individuals and of institutions that ostensibly could help them adjust, even thrive, despite these histories. These results make clear that universities must consider more effective ways to reconnect survivors with the university and create a culture where they feel supported and trusted. This safe environment could reduce both self-blame and stigma-threat motivated non-disclosure following the sexual assault, which are predictors of revictimization (Miller et al., 2007, 2011). Providing a range of supportive resources both within and outside of the university for survivors at different stages of healing could help to counteract previous negative experiences and reduce barriers to service use, improving connection and intuitional trust in the broader context.

In another domain, the elevated perceptions of risk that accompany this mistrust could have a lasting negative effect on well-being and overall uneasiness (Neilson et al., 2018; Pearson et al., 2021). To combat these effects, universities could consider ways to increase feelings of safety and boost survivors’ feelings of autonomy in ways that do not perpetuate harmful messages suggesting that individuals are responsible for preventing their own victimization. Ensuring that university administrators are trained effectively in how to empathetically respond to disclosures of sexual assault and provide meaningful support and useful and immediate resources while minimizing secondary wounding is a key step to cultivating a more positive environment. Informed by survivors’ perspectives, Walsh (2025) recommends that universities should seek to provide survivors with transparent options about available services, including reporting, increase the availability and quality of services, and improve communication between systems to increase institutional trust. Reflecting the importance of these institutional initiatives, interventions such as the Enhanced Access, Acknowledge, Act program have been successfully implemented by universities to empower women and reduce rates of sexual violence (Senn et al., 2017).

## Conclusion

This study shed light on the relationship between experiencing sexual assault prior to or during university and corresponding institutional trust and belonging, risk perceptions, and RMA. These findings add to a growing body of research that demonstrates the enduring influence that experiencing sexual assault can have on perceptions of institutions and their policies. Regardless of the context of their assault, survivors were more skeptical of institutions, reported greater perceived risk, and had higher levels of awareness of the broader cultural myths surrounding sexual assault. These findings have important implications for psychological outcomes of survivors and for understanding barriers to reporting and service use. Universities must first scrutinize the factors in place that perpetuate barriers for survivors, then establish new ways to foster institutional trust among survivors, create safer campus cultures through a range of supportive resources, and promote autonomy of all students in ways that boost feelings of safety.

## Supplemental Material

sj-docx-1-jiv-10.1177_08862605251363617 – Supplemental material for Taking Off the Rose-Colored Glasses: Experiences of Sexual Assault and Institutional Mistrust Supplemental material, sj-docx-1-jiv-10.1177_08862605251363617 for Taking Off the Rose-Colored Glasses: Experiences of Sexual Assault and Institutional Mistrust by Gabriella R. Petruzzello, Lucia F. O’Sullivan and Charlene F. Belu in Journal of Interpersonal Violence
