# Supplementary material for: Taking Off the Rose-Colored Glasses: Experiences of Sexual Assault and Institutional Mistrust
Source: J Interpers Violence. 2025 Aug 22;41(15-16):6063–88. doi: 10.1177/08862605251363617 (PMC13373291; doi:10.1177/08862605251363617)
Supplement: sj-docx-1-jiv-10.1177_08862605251363617 – Supplemental material for Taking Off the Rose-Colored Glasses: Experiences of Sexual Assault and Institutional Mistrust [file sj-docx-1-jiv-10.1177_08862605251363617.docx]

**Supplemental Materials: Exploratory Mediational Analyses**

**University Trust**

An exploratory mediation analysis using Hayes’ (2018) PROCESS macro (Model 4) was conducted. The model specified sexual assault history (X) as the independent variable (never, previous SA), perceptions of university policies (M) as the mediator, and campus belonging (Y) as the dependent variable (Figure 1). A bootstrap method with 5000 resamples was used to calculate the 95% confidence intervals (CI) for the indirect effect. The total effect of sexual history on campus belonging was significant, *b* = 0.12, *SE* = 0.04, *t*(1130) = 2.99, *p* = .003, [.04, .19], demonstrating that a history of sexual assault was associated with lower institutional trust. The direct effect of sexual assault victimization history on campus belonging was not significant after accounting for university trust, *b* = .01, *SE* = .04, *t*(1130) = .38, [-.06, .08]. To examine the mediation, the indirect effect of sexual assault history on campus belonging through institutional trust was tested. The bootstrapped 95% CI for the indirect effect of institutional trust on campus belonging was [0.07, 0.14], excluding zero, signifying that institutional trust significantly mediated the relationship between sexual assault experience and campus belonging.
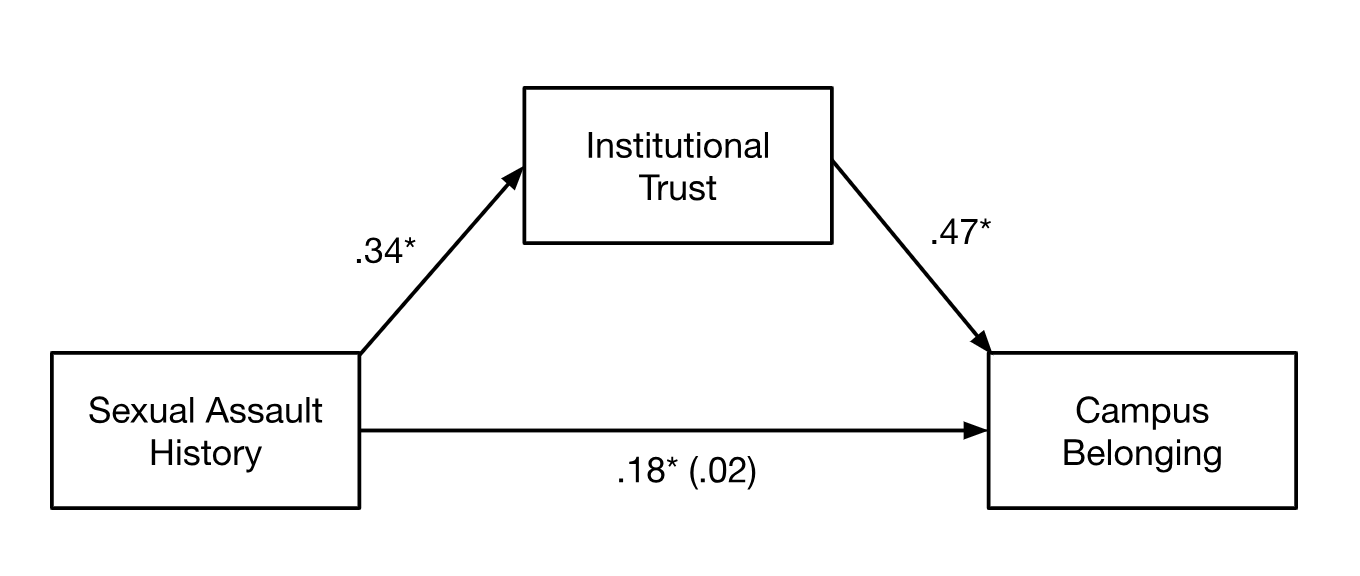


*Figure 1.* Standardized regression coefficients for the relationship between sexual assault history and campus belonging as mediated by institutional trust. The standardized regression coefficient between sexual assault history and campus belonging, controlling for institutional trust, is in parentheses.

**p* < .05

**Perceptions of University Policies**

For a second potential mediator under the auspices of institutional perceptions, the process was repeated with sexual assault history as the independent variable, perceptions of university policies as the mediator, and campus belonging as the dependent variable (Figure 2). The total effect of sexual assault history on campus belonging was significant, *b* = 0.12, *SE* = 0.04, *t*(1199) = 3.14, *p* =.002, [.05, .19], indicating that having experienced sexual assault was associated with lower campus belonging. The direct effect of sexual assault history on campus belonging, after accounting for perceptions of university policies, was not significant, *b* = 0.02, *SE* = 0.04, *t*(1199) = .46, *p* = .65, [-.05, .09]. The bootstrapped 95% CI for the indirect effect of perceptions of institutional policies on campus belonging was [0.07, 0.14], excluding zero, suggesting that perceptions of university policies significantly mediated the relationship between sexual assault experience and campus belonging. Overall, experiencing sexual assault was associated with lower campus belonging, due to more negative perceptions of the university’s sexual assault policies.


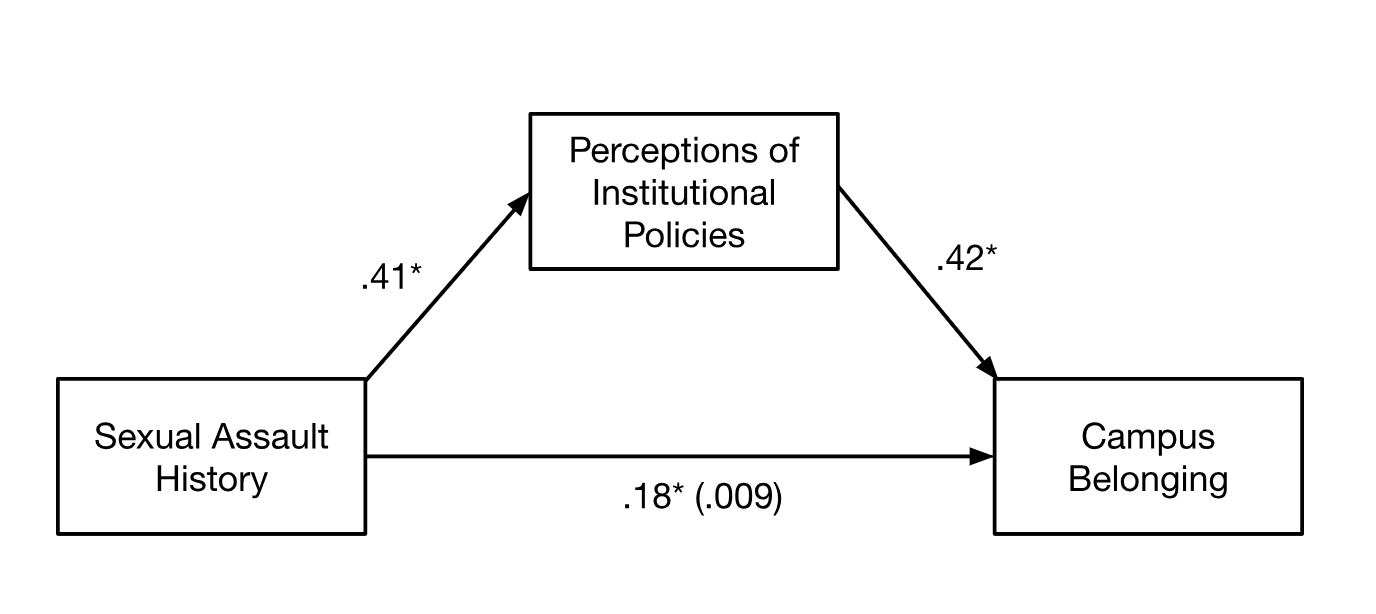


*Figure 2.* Standardized regression coefficients for the relationship between sexual assault history and campus belonging as mediated by perceptions of institutional policies. The standardized regression coefficient between sexual assault history and campus belonging, controlling for perceptions of institutional policies, is in parentheses.

**p* < .05

Hayes, A. F. (2018). *An Introduction to Mediation, Moderation, and Conditional Process*

*Analysis: A Regression-Based Approach* (2nd ed.). Guilford Press.

**Note:** In the mediational analyses above, the pre-university, during university, and both prior and during university groups are combined to increase statistical power. Results remain consistent (mediating effect of perceptions of institutional policies / institutional trust on belonging) when separate mediation analyses are conducted. For instance, there remains a mediational effect of institutional trust on belonging when only the pre-university group and no victimization history groups are compared and when only the two university groups and the no victimization history group are compared.
